# Is Drunkenness Curable

**Published:** 1892-01

**Authors:** 


### "IS DRUNKENNESS CURABLE?"

The public of late has been furnished with an abundance of literature touching the curability and non-curability of drunkenness. Obviously it is a matter of universal sociological importance. In the *North American Review*, for September, the subject is ably discussed by such eminent authorities as Dr. W. A. Hammond, Dr. T. N. Crothers, Dr. Cyrus Edson and Dr. E. N. Carpenter, and the views of these gentlemen, we believe, represent very accurately the views of the profession generally. As to the medical treatment of this diseased condition a certain method is being practiced considerably throughout this country, well known as the "Keeley Cure," which originated at Dwight, Illinois. Numerous "Keeley Institutes," modeled after the original, have been established in different cities.

The therapeutic agent employed is the bichloride of gold, hypodermically injected, four times daily for four or five weeks. The use of whisky during the course of treatment is not interdicted. This therapeutics is said by one of the regenerated to create a positive loathing for liquor. Dr. Keeley guarantees a cure of 95 per cent. of his cases, but seems willing to allow that 1 per cent. irretrievably fall from grace.

So much for the method and what is claimed for it.

Now we may say, without fear of successful refutation from the converts to the Keeley system, that there is no specific for drunkenness. It is a habit which may be so much indulged as to become almost one's normal state of existence. Like all other habits, it is not amenable to drug treatment. Drunkenness means a moral perversion, a weakening or complete loss of will power, a mental degeneration. To the poor victims who become slaves to the habit the craving for drink is too often absolutely irresistible. Under these circumstances, there is no drug in the pharmacopœia, or out of it, that can remove this insane thirst for alcohol. A patient and careful search for some such drug has been made since this ancient curse first afflicted humanity, and among the agents employed have been the tincture of capsicum, gentian, nitrate of strychnia, the bromides, etc. These, and others, have been tried and found wanting.

The newest remedy, so-called, is the double chloride of gold. Whisky is allowed *ad libitum*. Now, we believe this treatment to be irrational and unphysiological. It is a fundamental principle in the therapeutics of any disease, without exception, first of all things, to remove the cause, if it can be ascertained. The Keeley method disregards this entirely. Furthermore, the inebriate's nervous centres want and demand, in order for a cure to be effected, physiological rest, and an environment which places him in the most favorable condition for recovery. Manifestly these indications can best be obtained—only be obtained—in an institution especially equipped for this purpose. As Dr. Crothers puts it: "Drunkenness must be recognized as a disease legally, and the victim forced into conditions where he can live along the best sanitary lines of health; where medical treatment and control can be exact and perfect; and where physiological and hygienic training in its broadest and best sense can be applied."

Who can fully estimate the benefits to society, to morals and to civilization by promptly isolating such persons and keeping them in normal states of living?

The curability of the inebriate is far more certain than that of the insane. The liberty of both is equally dangerous: one is recognized; the other is seldom restrained until he becomes a criminal. The inebriate is mentally and physically sick, and needs the same help as the insane ; and the question of care is simply one of adequate means and remedies to reach the disease.

Only one word more. It is interesting. In the October number of the *Review*, above quoted, is a rejoinder from the pen of one of the apostles of this great benefactor, Dr. Keeley. Col. John Flavel Mims, LL. D., better known as "Felix Oldboy," was once numbered among the transgressors, and from his own confessions his case must have been, at one time, a desperate one. But his salvation was finally obtained, at Dwight, Illinois, in the "potent" influence of the impotent chloride of gold. He was reclaimed and redeemed, and with becoming gratitude, but unbecoming haste, he prepared the article to which we have alluded. We quote :

"No one who has not been similarly cursed with the disease of drink can know the joy of the moment in which my cure came to me as a fact. I do not believe—I know—that I am cured, and am satisfied as to its permanency. I do not understand the processes, but I know the fact. To-day I meet my fellow man with open gaze, knowing that I have conquered the black lion of the desert ; and my sense of freedom and happiness no man can paint."

Unfortunately, however, before this eloquent tribute from the pen of Felix Oldboy had been issued from the press, and therefore before it had been read by the public, this same Col. Mims, or what was left of him by a cruel irony of fate, was fished out of the gutter in New York and carried to Blackwell's Island, where he promptly died the drunkard's death.

---

We are indebted to Messrs. Lea & Shepard, of Boston, for their beautiful illustrated calendar, "All the Year Round." It is mounted with rings and chain, and makes an exceedingly neat and artistic design.

